# Loss of *BRCA1 *leads to an increase in epidermal growth factor receptor expression in mammary epithelial cells, and epidermal growth factor receptor inhibition prevents estrogen receptor-negative cancers in *BRCA1*-mutant mice

**DOI:** 10.1186/bcr2850

**Published:** 2011-03-11

**Authors:** Laura N Burga, Hai Hu, Ashish Juvekar, Nadine M Tung, Susan L Troyan, Erin W Hofstatter, Gerburg M Wulf

**Affiliations:** 1 Division of Hematology/Oncology, Beth Israel Deaconess Medical Center, 330 Brookline Avenue, Boston MA 02215, USA; 2 Cancer Biology Program, Beth Israel Deaconess Medical Center, Brookline Avenue, Boston MA 02215, USA; 3 Department of Surgery, Brigham and Women's Hospital, Boston, MA 02115, USA

## Abstract

**Introduction:**

Women who carry a *BRCA1 *mutation typically develop "triple-negative" breast cancers (TNBC), defined by the absence of estrogen receptor (ER), progesterone receptor and Her2/neu. In contrast to ER-positive tumors, TNBCs frequently express high levels of epidermal growth factor receptor (EGFR). Previously, we found a disproportionate fraction of progenitor cells in *BRCA1 *mutation carriers with EGFR overexpression. Here we examine the role of EGFR in mammary epithelial cells (MECs) in the emergence of *BRCA1*-related tumors and as a potential target for the prevention of TNBC.

**Methods:**

Cultures of MECs were used to examine EGFR protein levels and promoter activity in response to *BRCA1 *suppression with inhibitory RNA. EGFR was assessed by immunoblot and immunofluorescence analysis, real-time reverse transcriptase-polymerase chain reaction assay (RT-PCR) and flow cytometry. Binding of epidermal growth factor (EGF) to subpopulations of MECs was examined by Scatchard analysis. The responsiveness of MECs to the EGFR inhibitor erlotinib was assessed *in vitro *in three-dimensional cultures and *in vivo*. Mouse mammary tumor virus-Cre recombinase (MMTV-Cre) *BRCA1*^*flox/flox *^*p53*^+/- ^mice were treated daily with erlotinib or vehicle control, and breast cancer-free survival was analyzed using the Kaplan-Meier method.

**Results:**

Inhibition of *BRCA1 *in MECs led to upregulation of EGFR with an inverse correlation of *BRCA1 *with cellular EGFR protein levels (*r*^2 ^= 0.87) and to an increase in cell surface-expressed EGFR. EGFR upregulation in response to *BRCA1 *suppression was mediated by transcriptional and posttranslational mechanisms. Aldehyde dehydrogenase 1 (ALDH1)-positive MECs expressed higher levels of EGFR than ALDH1-negative MECs and were expanded two- to threefold in the *BRCA1*-inhibited MEC population. All MECs were exquisitely sensitive to EGFR inhibition with erlotinib *in vitro*. EGFR inhibition in MMTV-Cre *BRCA1*^*flox/flox *^*p53*^+/- ^female mice starting at age 3 months increased disease-free survival from 256 days in the controls to 365 days in the erlotinib-treated cohort.

**Conclusions:**

We propose that even partial loss of *BRCA1 *leads to an overall increase in EGFR expression in MECs and to an expansion of the highly EGFR-expressing, ALDH1-positive fraction. Increased EGFR expression may confer a growth advantage to MECs with loss of *BRCA1 *at the earliest stages of transformation. Employing EGFR inhibition with erlotinib specifically at this premalignant stage was effective in decreasing the incidence of ER-negative breast tumors in this mouse model.

## Introduction

Primary prevention of breast cancer has traditionally centered on estrogen receptor (ER) blockade, largely because the vast majority of breast cancers express ER and because ER antagonists are both easily administered and well-tolerated. However, ER antagonists do not prevent the most aggressive form of breast cancer: tumors that are ER- and progesterone (PR)-negative [1]. These tumors account for 15% to 20% of all breast cancers, occur with disproportionately high frequency in African-Americans and carry the worst prognosis [2,3]. The subgroup of women who are at highest risk for ER- and PR-negative breast cancers are women who carry a germline mutation in *BRCA1*. These women typically develop "triple-negative" breast cancers (TNBCs), which are defined by the absence of ER, PR and Her2 expression and are thought to be caused by genetic instability that results from a germline mutation in *BRCA1 *[4].

Though nominally classified as a diagnosis of exclusion (thus "triple-negative"), TNBC tumors frequently (72-75%) [5] overexpress epidermal growth factor receptor (EGFR), whereas only a minority (16%) of ER-positive breast cancers overexpress EGFR [5,6]. The high frequency of EGFR expression in TNBCs suggests that loss of *BRCA1 *may be coupled, either directly or indirectly, with EGFR overexpression in breast cancer [6]. This connection is further supported by the finding that sporadic TNBCs frequently exhibit both epigenetic silencing of *BRCA1 *[7] and overexpression of EGFR [5]. However, how TNBCs enrich for tumor cells with high EGFR expression is unknown.

Previously, we examined the proliferation and differentiation properties of *BRCA1*-mutant primary human MECs (hMECs) [8] and found a disproportionate fraction of progenitor cells in *BRCA1 *mutation carriers with concomitant EGFR overexpression and absence of ERα. Here we report that inhibition of *BRCA1 *in MECs leads to the upregulation of EGFR and the expansion of an aldehyde dehydrogenase 1 (ALDH1)-positive mammary epithelial progenitor cell population. We show that these MECs are exquisitely sensitive to EGFR inhibition with erlotinib and that EGFR inhibition *in vivo *could prevent the emergence of TNBCs.

## Materials and methods

### Reagents

Phycoerythrin (PE)-conjugated mouse anti-EGFR antibody (EGFR.1, 555997), PE-conjugated mouse immunoglobulin G2b (IgG2b) isotype control antibody (27-35; 555744) were obtained from BD Biosciences, San Diego, CA, USA, and QuantiBrite beads (340495) were obtained from BD Biosciences, San Jose, CA, USA. The ALDEFLUOR assay kit was purchased from STEMCELL Technologies, Durham, NC, USA. Rhodamine (Rh)-EGF (E-3481) was purchased from Invitrogen, Carlsbad, CA, USA. For immunofluorescence analysis, we used a mouse anti-EGFR antibody (EGFR.1, 555997) obtained from BD Biosciences, San Diego, CA, USA. For immunohistochemical analysis, we used anti-EGFR antibody (ab52894, rabbit monoclonal antibody EP38Y; Abcam, Cambridge, MA, USA), anti-ALDH1A1 antibody (ab52492, rabbit monoclonal antibody, EP1933Y; Abcam, Cambridge, MA, USA), anti-cleaved caspase 3 antibody (9661S, rabbit polyclonal antibody, Asp175; Cell Signaling Technology, Danvers, MA, USA), anti-Ki-67 antibody (9106-S, rabbit monoclonal antibody SP6; ThermoScientific, Fremont, CA, USA) and mouse anti-ERα antibody (MC-20, SC-524; Santa Cruz Biotechnology, Santa Cruz, CA, USA). For immunoblot analysis, mouse anti-*BRCA1 *antibody (MS110) was purchased from Calbiochem (manufactured by EMD Biosciences Inc., San Diego, CA, USA). Erlotinib was purchased from LC Laboratories (Woburn, MA, USA).

### Cell culture

Informed consent was obtained for the collection of primary hMECs from mastectomy specimens of *BRCA1 *mutation carriers (DFHCC-IRB legacy 04-405), and cells were isolated as described previously [8]. MECs were cultured in Mammary Epithelial Cell Growth Medium (MEGM; Lonza, Walkersville, MD, USA) or HuMEC medium (Gibco, Invitrogen, Carlsbad, CA, USA) supplemented with bovine pituitary extract. MCF-10A human epithelial cells (American Type Culture Collection (ATCC), Manassas, VA, USA), hMEC-expressing human telomerase reverse transcriptase (hTERT) cells and immortalized human mammary epithelial cells (HMLE cells) (gift from Dr. Robert Weinberg) were cultured in a mixture of Dulbecco's modified Eagle's medium-Ham's F-12 medium supplemented with 5% horse serum, 20 ng/ml EGF, 0.5 mg/ml hydrocortisone, 100 ng/ml cholera toxin and 10 μg/ml insulin. MCF-7 cells, the HCC1937 *BRCA1*-mutant breast cancer cell line (ATCC) and HCC1937 cells stably transfected with green fluorescent protein (GFP)-*BRCA1 *(gift from Dr. Ralph Scully) were kept in RPMI 1640 medium with 10% fetal bovine serum. For three-dimensional cultures, the cells were embedded in 40 μl of Geltrex (Invitrogen, Carlsbad, CA, USA) and cultured in eight-chamber culture slides (BD Falcon, San Diego, CA, USA).

### Cell viability and luciferase assays

For cell viability assays, MECs were seeded at a density of 250 cells/well in 96-well plates, and cell viability was determined using the CellTiter-Glo Luminescent Cell Viability Assay (Promega, Madison, WI, USA) according to the manufacturer's instructions, and absorption was read using a Wallac 3 plate reader. For luciferase assays, hMEC or MCF-7 cells were seeded into 24-well plates on day 1, transfected with BRCA1 small interfering RNA 1 (BRCA1 si1) or small interfering RNA 2 (BRCA1 si2) or control small interfering RNA (siRNA) on day 2 and with control or the full-length EGFR luciferase construct on day 3, followed by a luciferase assay performed on day 4. For each experiment, 2 μg of reporter construct were transfected in combination with either 1 ng of hMEC or 10 ng of Renilla thymidine kinase (Renilla TK) (MCF-7), and luciferase activity was determined using a Wallac 3 plate reader.

### Plasmids and inhibitory RNA constructs

The full-length EGFR promoter inserted 5' from a luciferase reporter [9] was a gift from Drs. Benjamin Purow and AC Johnson. The following sequences were used for the production of lentiviruses generating small hairpin RNA (shRNA): CAGCAGTTTATTACTCACTAA (*Brca1 *si1), CAGGAAATGGCTGAACTAGAA (*Brca1 *si2) and GCTAAACTCGTAATTCAACTT (scrambled control RNA interference (RNAi)). Transient transfection of siRNA was performed using siRNA and HyperFect transfection protocol (QIAGEN, Valencia, CA, USA) according to the manufacturer's instructions. Stably infected cells lines were produced using lentiviruses. The sh sequences were cloned into the pLKO.1 vector, and lentiviruses were produced in the 293FT cell line (Invitrogen, Carlsbad, CA, USA). The cells were infected and selected with puromycin as previously described [10].

### Flow cytometry

To measure the kinetics of binding of EGF, cells were grown for 24 hours in 6-cm dishes and serum-deprived for 4 to 6 hours at 37°C, followed by a 1-hour incubation on ice with indicated amounts of Rh-EGF. For uptake and binding, cells were incubated on ice with 10 ng of Rh-EGF, then the excess Rh-EGF was removed with an ice-cold phosphate-buffered saline (PBS) wash and the cells were incubated at 37°C for the indicated time intervals. The reaction was stopped on ice, and the noninternalized receptor was stripped with a light acid buffer (50 mM glycine, 150 mM NaCl, pH 3.0). The cells were gently dissociated with trypsin replacement TrypLE (Invitrogen, Carlsbad, CA, USA) and resuspended in PBS. The ALDEFLUOR assay kit was used to identify the stem and progenitor cell populations according to manufacturer's instructions. BODIPY aminoacetaldehyde (BAAA) was used as a substrate, and diethylaminobenzaldehyde was used as an inhibitor for negative controls. Cell surface-bound EGFR was measured using a phycoerythrin (PE)-conjugated EGFR antibody and PE-conjugated mouse IgG2b isotype control antibody. Following gentle cell dissociation or ALDEFLUOR assay, the cells were washed, resuspended in 80 μl of PBS with bovine serum albumin (BSA) or ALDEFLUOR assay buffer and 20 μl of either antibody or isotype control solution were added. Reactions were incubated on ice for 30 minutes, the cells were washed with either PBS and BSA or ALDEFLUOR assay buffer and resuspended in 0.5 ml of PBS or ALDEFLUOR assay buffer. QuantiBrite beads were used to estimate the number of EGFR molecules per cell. Samples were measured using a FACSAria™ II Cell Sorter 5-laser SORP instrument (BD Biosciences, San Jose, CA, USA) or sorted using a MoFlo sorter (Beckman-Coulter, Inc, Miami FL, USA).

### Immunofluorescence

Cells cultured on coverslips for 24 hours were fixed for 10 minutes at room temperature in 3% paraformaldehyde/2% sucrose solution, rinsed twice with PBS and permeabilized with ice-cold Triton X-100 solution (0.5% Triton X-100, 20 mM HEPES ((4-(2-hydroxyethyl)-1-piperazineethanesulfonic acid )), pH 7.4, 50 mM NaCl, 3 mM MgCl_2_, 300 mM sucrose) for 3 minutes on ice. The cells were rinsed for 5 times with PBS and blocked for 20 minutes with 10% goat serum followed by incubation with primary antibody anti-EGFR (EGFR.1) and anti-ALDH1A1 (EP1933Y) for 20 minutes at 37°C. Cells were washed two times and incubated for 20 minutes at 37°C with secondary antibody Alexa 488-conjugated anti-rabbit or Alexa 594-conjugated anti-mouse antibody (1:1,000 dilution; Invitrogen). The nuclei were stained with DAPI (1:10,000 dilution; 4',6-diamidino-2-phenylindole), and the slides were examined using a Nikon fluorescence microscope (Nikon, Tokyo, Japan). For quantification of the fluorescence signal, the mean intensity was determined using ImageJ software in four different fields for each sample. Experiments were performed in triplicate, and the means and standard deviations of the signal intensities were calculated for each condition.

### Real-time RT-PCR

Total RNA was extracted using the RNeasy Plus Mini Kit (QIAGEN). RNA was reverse-transcribed using the AccuScript enzyme in the AccuScript High Fidelity RT-PCR System (Agilent Technologies, Stratagene Products Division, La Jolla, CA, USA). A quantitative real-time RT-PCR assay was carried out on a Rotor-Gene 6000 cycler (Corbett Life Science, San Francisco, CA, USA) using SYBR Green Supermix (Bio-Rad Laboratories, Hercules, CA, USA). The PCR reaction (15 μl) was performed under the following conditions: 95°C for 10 minutes followed by 45 cycles at 95°C for 20 seconds, at 56°C for 25 seconds and at 72°C for 40 seconds. The expression of the *EGFR *gene was normalized to GAPDH (glyceraldehyde 3-phosphate dehydrogenase) levels. The primer sequences for human EGFR cDNA (70 bp) were forward primer 5'-GCACCTACGGATGCACTGG-3' and reverse primer 5'-GGCGATGGACGGGATCTTA-3'.

### Immunohistochemistry, morphometry and statistics

Immunohistochemistry was performed as described previously [11]. Scoring for EGFR expression was done according to the following system: Score 0 no staining or staining in less than 10% of cells. Score 1+, a faint perceptible membrane staining can be detected in more than 10% of cells. Score 2+, a weak to moderate complete membrane staining is observed in more than 10% of cells. Score 3+, a strong complete membrane staining is observed in more than 10% of the cells. Colonies were documented using ACT-1 software connected to an Olympus SZX12 or a Nikon EclipseS100 microscope and analyzed using SIGNATURE software [12].

A two-sided *t*-test was used to determine statistical significance. Kaplan-Meier analysis was done using the GraphPad Prism software package (GraphPad Software, La Jolla, CA, USA), and survival statistics were calculated using the log-rank test. Scatchard analysis of Rh-EGF binding was done as described previously [13,14].

### Animal experiments

All animal experiments were conducted in accordance with Institutional Animal Care and Use Committee-approved protocols. Experimental female mice, *Brca1*^*flox/flox*^, MMTV-Cre and *p53*^+/-^, were obtained by breeding *Brca1 *conditional knockout mice from the National Institutes of Health repository (01XC8, strain C57BL/6), originally generated by Xu *et al. *[15], who made these mice available to us via the National Cancer Institute repository, with MMTV-Cre mice (B6129-TgN(MMTV-Cre)4Mam; Jackson Laboratory, Bar Harbor, ME, USA) [16] and *p53*-knockout mice (P53N12-M, C57BL/6; Taconic Farms, Germantown, NY, USA) [17]. At the time of the study, the mice had been inbred for 2 years (seven generations). The floxed or wild-type status of *Brca1*, the presence of the MMTV-Cre transgene and *p53 *heterozygosity were determined by PCR as previously described [15]. Mice were examined for the occurrence of tumors twice weekly. When tumor metrics were performed, the length and width of the tumor were determined using calipers and the tumor volume was determined by calculating width^2 ^× length/2. Tumor growth was recorded as the ratio of tumor growth to tumor volume at the time of diagnosis.

## Results

### *BRCA1 *inhibition results in increased EGFR expression

**Figure 1:**
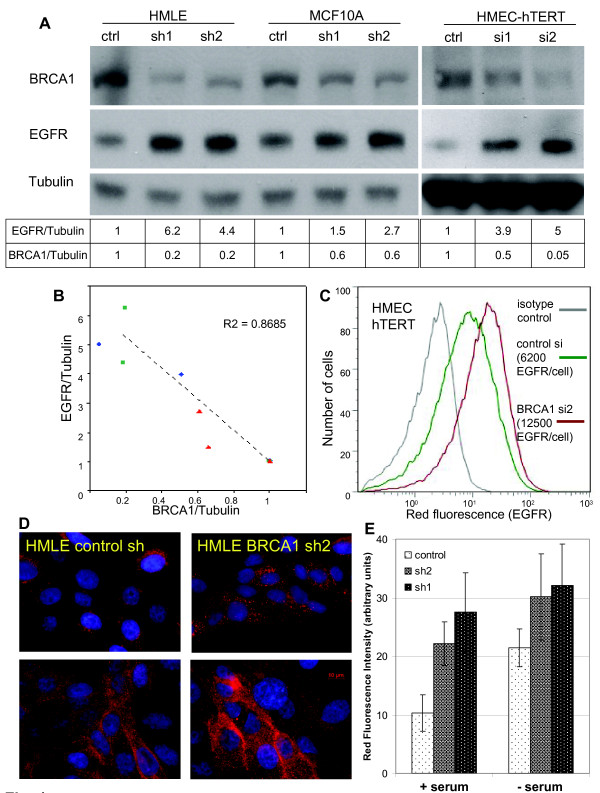
***BRCA1 *suppression in mammary epithelial cells (MECs) leads to an increase in epidermal growth factor receptor (EGFR) expression**. **(A) **MECs were transfected with *BRCA1 *control or small interfering RNA 1 or 2 (si1, si2), or they were infected with lentivirus-expressing control or *BRCA1*-specific small hairpin RNA (sh1, sh2) and lysed for immunoblot analysis.. **(B) **The intensities of the chemiluminescence signals of EGFR, *BRCA1 *and tubulin levels were quantified using ImageJ software. **(C) **Flow cytometry using phycoerythrin-conjugated anti-EGFR antibodies shows an increase in cell surface EGFR expression after *BRCA1 *suppression (hMEC-hTERT; similar results were obtained with MCF-10A cells). **(D) **Immunofluorescence of EGFR in asynchronously growing HMLE (top) and after serum deprivation (bottom) in control (left) and *BRCA1*-suppressed MECs (right). Experiments were performed in triplicates using controls and two different small hairpin-containing MEC lines. **(E) **The fluorescence intensity of the images was quantified using ImageJ software.

To examine whether EGFR upregulation is directly related to the loss of *BRCA1*, we suppressed *BRCA1 *in different MEC lines, including MCF-10A [18], hMEC-hTERT and HMLE [19]. These MEC lines have not yet undergone transformation, and instead are propagated as immortalized cells. hMECs were transfected with control or *BRCA1*-directed siRNA and analyzed 72 to 120 hours after transfection. MCF-10A and HMLE cells showed poor transfection efficiency upon transient transfection with siRNA, and therefore these cells were infected with lentiviruses that expressed shRNAi against *BRCA1 *(Figure 1A) and selected for pools of infected cells with puromycin. Asynchronously growing cells were lysed and analyzed for EGFR expression. Throughout these experiments, the effects observed after short-term suppression of BRCA1 with transient transfection in hMECs were similar to the results obtained in MCF-10A and HMLE cells with longer-term suppression of BRCA1 after lentiviral infection and puromycin selection.

In all three cell lines and with either approach, we found that EGFR protein levels as measured by immunoblotting with anti-EGFR antibodies increased when *BRCA1 *was inhibited (Figure 1A). We measured the density of the immunoblotting signals and found that, with *BRCA1 *inhibition, EGFR levels increased by up to five times over baseline (Figure 1A). In addition, there appeared to be a tight negative correlation of *BRCA1 *and EGFR levels (*r*^2 ^= 0.87), suggesting a regulatory role of *BRCA1 *for EGFR (Figure 1B). Next, we examined EGFR levels in response to *BRCA1 *suppression under conditions of steady-state growth or serum starvation using immunofluorescence and quantification of the EGFR fluorescence signal (Figure 1D, bar graph). We found that BRCA1 inhibition led to EGFR upregulation under both conditions, as well as asynchronous growth and starvation, suggesting that the effect of *BRCA1 *suppression on EGFR expression is not mediated by the absence or presence of growth factors (Figure 1D).

We then used flow cytometry to examine whether the increase in total cellular EGFR protein was accompanied by an increase in EGF binding sites on the cell surface as opposed to intracellular accumulation. We found that hMEC-hTERT expressed an average of 6 × 10^3 ^EGFR per cell, which increased up to twofold after siRNA inhibition of *BRCA1 *(Figure 1C). A similar increase of cell surface EGFR was seen with a second BRCA1-targeted siRNA (si1) in hMECs and using BRCA1-directed shRNA in MCF-10A cells (Figures 4G and 4H). Immunofluorescence of EGFR using anti-EGFR antibodies in hMEC-hTERT confirmed that *BRCA1 *inhibition resulted in an increase in both surface and intracellular EGFR, with a strong increase of EGFR on the cell surface upon serum deprivation after *BRCA1 *inhibition (Figure 1D). In summary, we found that both transient and stable suppression of *BRCA1 *led to an up to fivefold increase in EGFR protein and to an approximately twofold increase in the number of EGFR expressed on the MEC surface. Thus, the increase in intracellular EGFR was more pronounced than the increase in cell surface-expressed EGFR upon *BRCA1 *inhibition.

### *BRCA1 *inhibition increases EGFR expression through both an increase in transcription as well as stabilization of the EGFR protein

**Figure 2:**
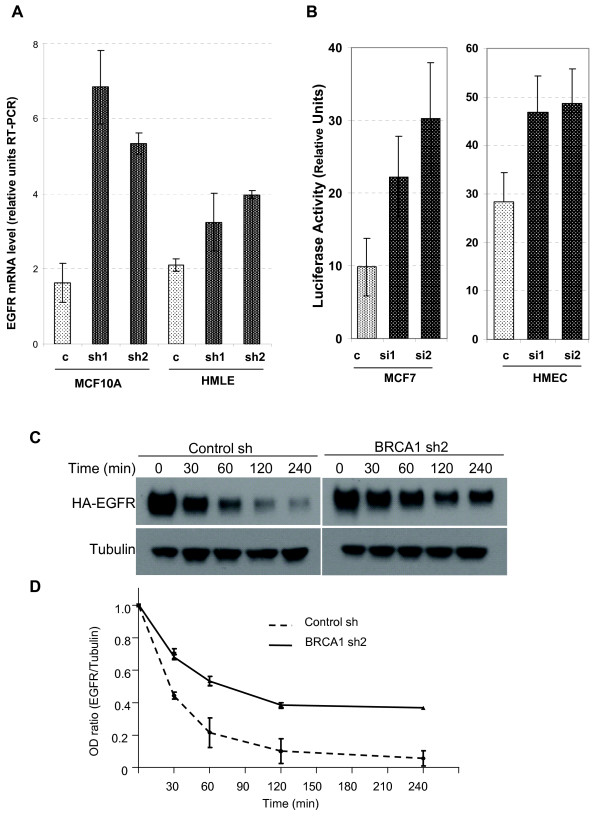
**Transcriptional and posttranslational mechnisms lead to an in crease in EGFR expression after *BRCA1 *inhibition**. **(A) **EGFR mRNA levels were determined in control MECs (light gray bars) and in mammary epithelial cells (MECs) expressing small hairpin RNA directed against *BRCA1 *(dark bars). RNA levels were normalized for GAPDH (glyceraldehyde 3-phosphate dehydrogenase) expression. RT-PCR, real-time reverse transcriptase-polymerase chain reaction; HMLE, human mammary epithelial cells. **(B) **Decreased EGFR promoter activity as a result of short-term BRCA1 suppression in MECs and MCF-7 cancer cells. BRCA1 control and small interfering RNA (siRNA) and the full-length EGFR promoter were transfected as indicated, and luciferase activity was normalized for Renilla thymidine kinase expression. **(C and D) **EGFR half-life increases from less than 30 minutes to over 70 minutes after *BRCA1 *inhibition. Control and *BRCA1 *sh2-expressing MCF-10A cells were transfected with hemagglutinin-tagged EGFR 48 hours prior to the time course, serum-deprived for 8 hours, then treated with cycloheximide at 100 μg/ml for 2 hours, and stimulated with epidermal growth factor at point 0. Lysates were prepared and immunoblotted at the indicated time points. The chemiluminescence signal was quantified as in Figure 1B. Similar results were obtained with BRCA1 sh1-expressing cells. In D the ratio of the optical density (OD) for EGFR to Tubulin is plotted against time.

We next examined the molecular mechanisms by which *BRCA1 *inhibition caused an increase in EGFR protein. Given earlier reports that *BRCA1 *can function as a transcriptional regulator and that it specifically regulates another receptor tyrosine kinase, insulin-like growth factor I receptor (IGF-IR) [20,21], we analyzed mRNA levels using quantitative RT-PCR. We found that in MEC lines with stably suppressed *BRCA1 *levels, EGFR mRNA was upregulated 1.5- to twofold in HMLE and two- to threefold in MCF-10A cells, indicating an increase in EGFR transcription in response to *BRCA1 *downregulation (Figure 2A). We next examined the effects of BRCA1 suppression on EGFR promoter activity to determine whether the increase in EGFR mRNA was due to direct transcriptional activation. As these luciferase assays required transient transfection of siRNA and reporter plasmid, they could be performed only in hMECs, not in MCF-10A or HMLE cells. Therefore, we performed a second set of luciferase assays in MCF-7 breast cancer cells. We found that EGFR promoter activity increased up to twofold upon BRCA1 suppression (Figure 2B), consistent with the increase in mRNA levels observed (Figure 2A) and confirming that *BRCA1 *exerts a negative regulatory role on EGFR transcription.

Because *BRCA1 *also has ubiquitin ligase activity toward tubulin [22], ERα [23] and phosphorylated Akt [24], and because we observed a pronounced increase in intracellular EGFR upon *BRCA1 *suppression (Figure 1D), we tested whether *BRCA1 *suppression affects EGFR stability after blockade of protein biosynthesis with cycloheximide (Figures 2C and 2D). Interestingly, *BRCA1 *suppression increased the half-life of the EGFR protein from less than 30 minutes to over 75 minutes (Figure 2D). Thus, there appear to be at least two mechanisms that result in an increase in EGFR protein levels upon *BRCA1 *suppression, transcriptional regulation and protein stabilization.

### ALDH1-positive cells show an increase in EGFR expression

**Figure 3:**
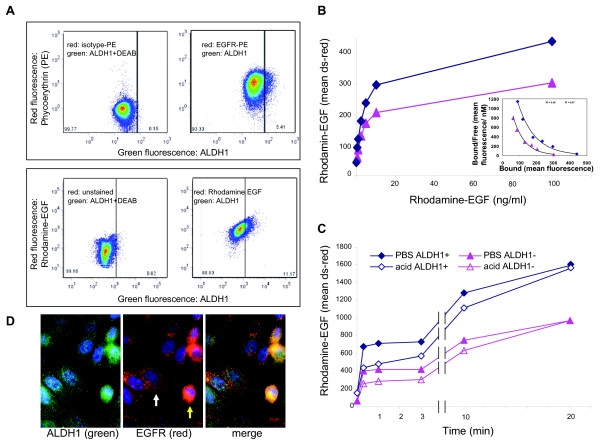
**Heterogeneity and kinetics of epidermal growth factor receptor (EGFR) expression in mammary epithelial cells (MECs)**. **(A) **Dual staining of aldehyde dehydrogenase 1 (ALDH1)-positive cells (green fluorescence) with anti-EGFR antibodies (top) or rhodamine (Rh)-labeled epidermal growth factor (EGF) (bottom) in human MEC-human telomerase reverse transcriptase (hMEC-hTERT). Negative controls used to adjust compensation settings are shown (left). ALDH1-positive cells show higher EGF binding and a higher number of EGF receptors (right). DEAB, diethylaminobenzaldehyde. **(B) **Binding of EGF in ALDH1-negative or ALDH1-positive MECs. Cells were incubated at 4°C with the indicated amounts of EGF and labeled with ALDH1 reagent. Inset: Scatchard analysis of EGF binding. *K*_d _values were 0.32 nM for both the ALDH1^+ ^and ALDH1^- ^fractions. The intensity of the red fluorescence was measured using the channel for red fluorescence (Discosoma Red - ds-red) of the flow cytometer. **(C) **EGF binding and uptake. Cells were incubated with 10 ng/ml Rh-EGF at 4°C, free Rh-EGF was removed and MECs were counterstained with ALDH1 reagent. To assess binding and uptake, cells were washed with phosphate-buffered saline (PBS) (solid symbols). To assess solely uptake, bound EGF was removed using an acetic acid wash (bordered symbols). **(D) **Immunofluorescence of EGFR and ALDH1 in aysynchronously growing MECs (human MEC-human telomerase reverse transcriptase).

**Figure 4:**
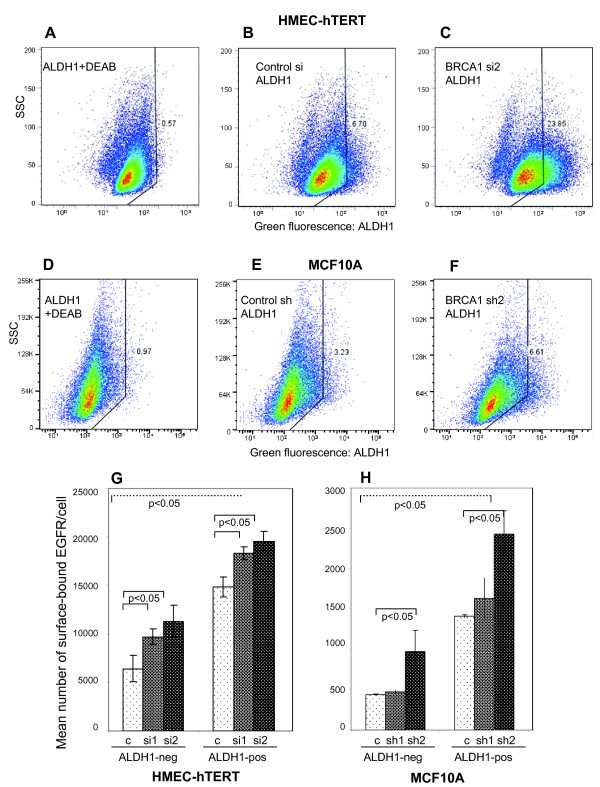
***BRCA1 *inhibition increases EGFR expression in the aldehyde dehydrogenase 1 (ALDH1)-negative and -positive MEC fractions**. Cells were **(A, B and C) **transfected with either control or *BRCA1 *small interfering RNA (siRNA) or **(D, E and F) **infected with lentiviruses expressing control or *BRCA1 *small hairpin RNA (shRNA). Cells treated with the ALDH1 inhibitor diethylaminobenzaldehyde (DMEA) served as controls **(A and D)**. **(G and H) ***BRCA1 *inhibition increases the expression of cell surface-bound EGFR in human MEC-human telomerase reverse transcriptase (hMEC-hTERT) or MCF-10A cells in the ALDH1-negative and the ALDH1-positive fractions. Live cells were harvested and incubated with ALDH1 reagent followed by immunostaining with phycoerythrin (PE)-conjugated anti-EGFR antibodies and quantification of EGFR using the QuantiBrite standards for PE.

Using immunofluorescence imaging, we noted heterogeneity with regard to EGFR expression in both control MECs as well as in MECs after *BRCA1 *inhibition (Figures 1D and 3D). An increased expression of EGFR in basal cells was previously observed in murine MECs [25] and hMECs [26], and a drift toward high EGFR expression was seen in cell line models of basaloid breast cancer [27], which led us to examine whether the EGFR levels differed between stem and non-stem cells as defined by the expression of ALDH1 [28,29]. We found that mean numbers of EGFR were higher in the ALDH1-positive fractions of MECs than in the ALDH1-negative fractions. (Figure 3A, top, and Figure 4G). Consistently, ALDH1-positive MECs showed an increased binding of Rh-labeled EGF when compared to the ALDH1-negative fraction (Figure 3A, bottom, and Figures 3B and 3C). Given these differences in cell surface-expressed EGFR, we compared the kinetics of EGF binding and internalization between ALDH1-positive and ALDH1-negative MECs. For the binding assay, cells were incubated with increasing concentrations of Rh-labeled EGF, and binding was analyzed using flow cytometry (Figure 3B). Scatchard analysis of Rh-EGF binding at 4°C showed that both the ALDH1-positive and ALDH1-negative population bound EGF with similar affinity (*K*_d _= 0.32 nM) (Figure 3B, inset). For binding and internalization (Figure 3C), cells were preincubated with Rh-EGF at 4°C to allow for binding, followed by removal of unbound Rh-EGF incubated for the indicated times and concentrations with Rh-labeled EGF at 37°C, and then washed with either PBS or an acidified buffer as described previously [30], followed by ALDH1 staining. While the PBS wash removes only unbound Rh-EGF, the acidified buffer removes both receptor-bound and receptor-unbound EGF, that is, fluorescence after the acidic wash is representative of internalized EGF. We found that EGF binding was biphasic, both in ALDH1-positive and ALDH1-negative cells, with an initial saturation of EGF binding sites after 5 minutes, followed by a second, slower phase of binding and internalization. Internalization was complete after 30 minutes at 37°C (Figure 3C).

In summary, binding and internalization kinetics were similar in ALDH1-positive and ALDH1-negative MECs, while the total number of circulating EGF receptors was increased in the ALDH1-positive fraction.

### *BRCA1 *inhibition increases EGFR expression in both the ALDH1-negative and the expanded ALDH1-positive cell pool

The heterogeneity of the MEC pool, and how this heterogeneity is affected by the loss of *BRCA1*, is an area of active research [29,31]. Several immunophenotype profiles have been used to define MEC progenitor cells, such as the CD24^low^/CD44^high ^profile [32] and the CD49f^+^/EpCam^+ ^profile [31]. Consistent with the data published by Liu *et al. *[29] and with our own observations in *BRCA1 *mutation carriers [11], we found that the percentage of ALDH1-positive cells increased fourfold in hMEC-hTERT and doubled in MCF-10A cells in response to inhibition of *BRCA1 *(Figures 4C and 4F). In addition, we found a corresponding increase in the CD24^low^/CD44^high ^population in both HMLE and MCF-10A cells expressing BRCA1 shRNA (Additional file 1, Figure S1), thus confirming an increased MEC progenitor cell pool in response to BRCA1 inhibition [29,32]. Using two-color flow cytometry and QuantiBrite beads [33], we found that ALDH1-positive MECs carried two to three times the number of EGF receptors compared with ALDH1-negative cells (Figures 3A, 4G and 4H). Upon *BRCA1 *inhibition with siRNA in hMECs or with shRNA in MCF-10A cells, a significant increase of EGFR was observed in ALDH1-negative and ALDH1-positive MECs (Figures 4G and 4H, dark bars). Thus, our data show that *BRCA1 *inhibition affects EGFR expression in two ways: *BRCA1 *suppression leads to the expansion of the highly EGFR-expressing ALDH1-positive MEC pool (Figures 4C and 4F through 4H), and, second, *BRCA1 *inhibition raises the numbers of EGF receptors per cell in all MECs (ALDH1-positive and ALDH1-negative cells), likely through transcriptional activation (Figure 2A) and posttranslational mechanisms (Figure 2B).

### EGFR inhibitor erlotinib blocks the outgrowth of normal and *BRCA1*-deficient MECs

**Figure 5:**
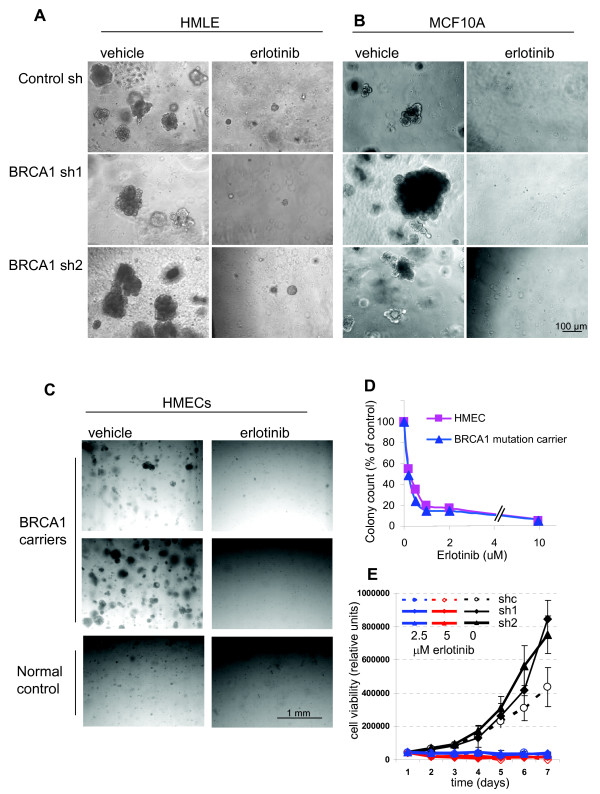
***BRCA1 *inhibition leads to increased colony formation and size that is completely blocked by epidermal growth factor receptor (EGFR) inhibition**. Mammary epithelial cells (MECs) expressing either **(A and B) **control small hairpin RNA (shRNA) or *BRCA1*-directed shRNA or **(C and D) **primary MECs isolated from reduction mammoplasties or *BRCA1 *mutation carriers were seeded and photographed after 10 days in culture. All colony formation was completely suppressed when cells were grown in the presence of erlotinib at 2 μM shown in phase images for each cell type (right). **(D) **MEC growth is inhibited at concentrations as low as 0.5 μM erlotinib. Cells were seeded at 5,000 cells/well, allowed to grow for 10 days in the presence of the indicated amounts of erlotinib and then counted. **(E) **Cell viability assay of control and BRCA1 shRNA-expressing human MECs. Cells were seeded in triplicate in 96-well plates at 250 cells/well, and cell viability was determined daily. Dotted line and open symbol represent control cells, and closed symbols represent cells expressing BRCA1-directed shRNA. Black lines, vehicle control; blue lines, culture in the presence of 2.5 μM erlotinib; red line, culture in the presence of 5 μM erlotinib.

**Figure 6:**
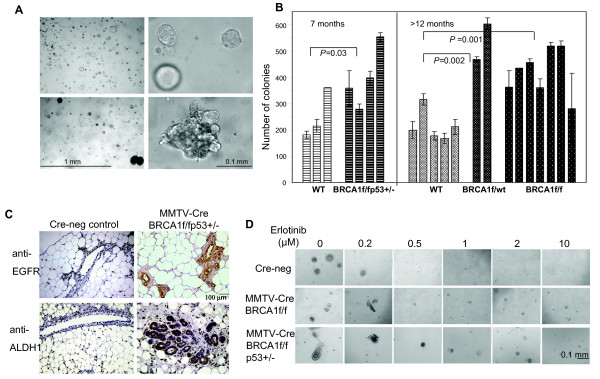
**Proliferation and differentiation properties of MECs from MMTV-Cre BRCA1-mutant mice . (A and B) **Growth and proliferation properties of mammary epithelial cells (MECs) isolated from mouse mammary tumor virus-Cre recombinase (MMTV-Cre) *BRCA1*-mutant mice are similar to MECs isolated from human *BRCA1 *mutation carriers. MECs were harvested and plated as described previously [8]. **(A, top) **Cultures from wild-type (WT) control mice resulted in round acinar structures, whereas **(A, bottom) **cultures from MMTV-Cre *BRCA1*^*flox/flox *^*p53*^+/- ^mice showed complex and irregular features. **(B) **The overall colony-forming efficiency of murine *BRCA1*-mutant MECs is increased. Non-tumor-bearing WT and MMTV-*BRCA1*^*flox/flox *^*p53*^+/- ^MECs were compared at age 7 months, and non-tumor-bearing mice from MMTV-Cre *BRCA1*^*flox/WT *^(heterozygous loss of *BRCA1*) MMTV-Cre *BRCA1 *^*flox/flox *^were compared at age 12 to 13 months. **(C) **Mammary glands from MMTV-Cre BRCA1 ^*flox/flox *^*p53*^+/- ^mice contain epidermal growth factor receptor (EGFR) and aldehyde dehydrogenase 1 (ALDH1)-positive acini. Immunohistochemistry for EGFR and ALDH1 was performed on five *BRCA1*-mutant mammary glands and seven Cre-negative controls. Representative images are shown at × 20 original magnification. **(D) **Erlotinib is active in suppressing the growth of murine MECs from mice of WT, MMTV-Cre *BRCA1 *^*flox/flox *^or MMTV *BRCA1 *^*flox/flox *^*p53*^+/- ^background. All MECs were seeded in Matrigel-based cultures and photographed and analyzed for colony formation using SIGNATURE software [12] after 14 days of culture.

Given our findings of EGFR upregulation in MECs upon *BRCA1 *inhibition, as well as our previous findings of altered growth and differentiation patterns of EGFR-expressing MECs isolated from *BRCA1 *mutation carriers [8], we asked whether EGFR inhibition could block this phenotype. First, we examined the growth characteristics of control and *BRCA1*-suppressed or *BRCA1*-mutant MECs. Consistent with our previous data, we found that after experimental suppression of *BRCA1*, MECs formed larger colonies with greater efficiency than control cells in the three-dimensional Matrigel-based cultures (Figures 5A and 5B). Similar findings were obtained with primary MECs from *BRCA1 *mutation carriers, which yielded a higher number of larger colonies than controls (Figure 5C) [8]. Finally, our results were further confirmed by MEC cultures from MMTV-Cre *BRCA1*^*flox/flox *^mice, in which even the heterozygote loss of *BRCA1 *led to increased clonality of MECs (Figure 6B). Thus, our data in primary hMECs, murine MECs and immortalized MECs with experimental *BRCA1 *suppression all confirm that even partial suppression or heterozygote loss of *BRCA1 *causes an increase in the clonogenicity and proliferative potential of MECs.

Next, we treated MECs with the EGFR inhibitor erlotinib and found that erlotinib efficiently blocked the outgrowth of colonies from all MECs, controls as well as *BRCA1*-suppressed MECs (Figures 5A and 5B), of normal and *BRCA1*-mutant primary MECs (Figure 5C), as well as of murine MECs that were wild-type or *BRCA1*-deficient (Figure 6C). In dose-response experiments, we found that <1 μM erlotinib was sufficient to suppress MEC outgrowth in both hMECs (Figure 5D) and murine MECs (Figure 6C), indicating that MECs carrying a wild-type EGFR are highly sensitive to the growth-inhibitory effect of erlotinib. In addition, we used a 3-(4,5-dimethylthiazol-2-yl)-2,5-diphenyltetrazolium bromide (MTT)-based cell viability assay to determine the effects of erlotinib on MEC growth. Cells were seeded at equal densities, and cell viability was measured daily over a period of 7 days (Figure 5E). This quantitative cell viability assay confirmed that both cell types that expressed *BRCA1*-inhibitory shRNA grew significantly faster and reached double the cell number after 7 days of culture compared to controls (Figure 5E), thus confirming that loss of *BRCA1 *leads to accelerated proliferation of MECs. In the quantitative cell viability assay, MECs with loss of *BRCA1 *were equally as sensitive to erlotinib as wild-type cells (Figure 5E), confirming our observations in the colony formation assays (Figures 5A through 5D and Figure 6). In summary, both readout methods, colony formation assay as well as cell viability assay, confirmed that MECs with loss of *BRCA1 *that express higher EGFR levels proliferate more rapidly than controls and that this increase in proliferation remains highly sensitive to the growth-inhibitory effect of erlotinib.

### MECs from *BRCA1*-mutant mice show proliferation and differentiation patterns similar to MECs from human *BRCA1 *mutation carriers

The model of MMTV-Cre flox-directed deletion of *BRCA1 *was first developed by Xu *et al. *[15] and has been used extensively to examine *BRCA1*-related tumorigenesis. When grown in three-dimensional Matrigel-based cultures, murine MECs grew in patterns similar to those of hMECs, that is, cells from wild-type mice formed hollow acini after 10- to 14-day culture periods (Figure 6A, top). In cells isolated from MMTV-Cre *BRCA1*^*flox/flox *^p53^+/- ^mice, we found large, complex, solid structures (Figure 6A, bottom), similar to those that we found in human *BRCA1 *mutation carriers [8]. Next, we examined the mammary gland tissues of five MMTV-Cre *BRCA1 *^*flox/flox *^*p53*^+/- ^mice and seven Cre-negative, age-matched control mice for the expression of EGFR and ALDH1 (Figure 6C). We found that the mammary glands of *BRCA1*-mutant mice in general contained more acini than the controls. In each of the MMTV-Cre *BRCA1 *^*flox/flox *^p53^+/- ^mammary glands, we found entire acini that stained positive for both EGFR and ALDH1, while only occasional single cells were positive in any of the Cre-negative control glands.

MMTV-Cre *BRCA1 *^*flox/flox *^p53^+/- ^mice develop breast cancer with a latency of about 8 to 10 months, while MMTV-Cre *BRCA1 *^*flox/*^^flox ^mice develop tumors with relatively low penetrance beyond age 1 year or older [34], and MMTV-Cre *BRCA1 *^*flox*^/wt mice rarely develop spontaneous breast cancers. Therefore, we examined the clonogenicity of murine MECs that had not yet formed tumors at age 7 months for MMTV-Cre *BRCA1 *^*flox/flox *^p53^+/- ^mice and at age 12 months for MMTV-Cre *BRCA1 *^*flox/flox *^or MMTV-Cre *BRCA1 *^*flox*^/wt. In comparison to wild-type cells, all three mutant cell types showed significantly increased colony formation (Figure 6B). Interestingly, this increase in clonogenicity was observed not only in cells from mice with homozygotic loss of *BRCA1 *but also in cells from mice with heterozygotic loss of *BRCA1 *(MMTV-Cre *BRCA1 *^*flox*^/wt), indicating that loss of a single allele, which is a situation analogous to a human *BRCA1 *mutation carrier, leads to an increase in colony formation (Figure 6B).

Next, we examined whether treatment with erlotinib was similarly effective in murine MECs as it was in hMECs, and we found that colony formation was suppressed effectively at 1 μM erlotinib in the medium (Figure 6D). On the basis of these findings, we tested the efficacy of erlotinib for the primary prevention of breast cancer in *BRCA1*-mutant mice.

### EGFR inhibitor erlotinib prevents the development of ER-negative, but not of ER-positive, breast cancers in *BRCA1*-mutant mice

**Figure 7:**
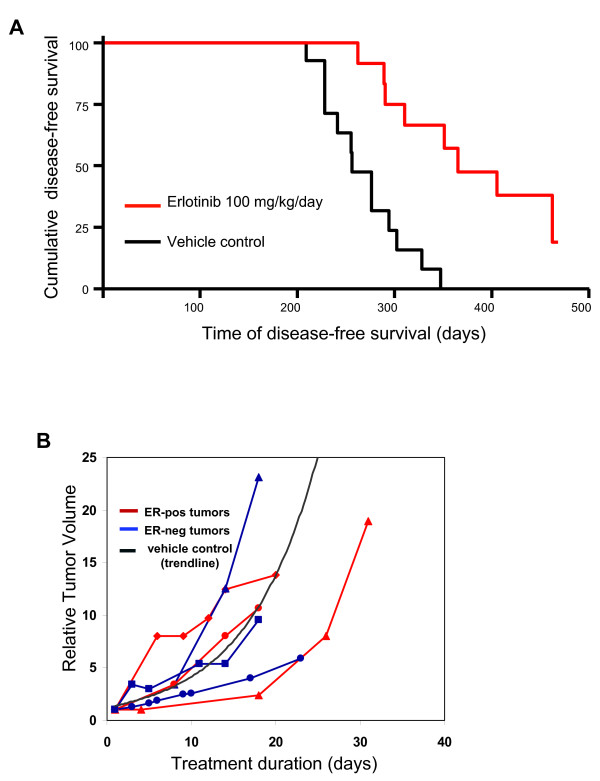
**Erlotinib as a chemopreventative agent in (MMTV-Cre) BRCA1 ^flox/flox ^p53^+/- ^mice**. **(A) **Erlotinib prevents the emergence of estrogen receptor-negative (ER^-^) but not estrogen receptor-positive (ER^+^) breast cancers in mouse mammary tumor virus-Cre recombinase (MMTV-Cre) *BRCA1 *^*flox/flox *^*p53*^+/- ^mice. Virgin female mice were treated as controls or with 100 mg/kg erlotinib via oral gavage once daily, seven days per week. Tumors were recorded when they were first palpated. Kaplan-Meier graphing and analysis of disease-free survival were performed using the GraphPad Prism software package. **(B) **The growth of established breast cancers is not affected by erlotinib treatment. Mice that developed tumors in the control cohort were switched to erlotinib treatment, and the tumor volume relative to the tumor volume at diagnosis was plotted against treatment time. ER status was determined after necropsy. The trend line for vehicle control-treated tumors was established on the basis of the tumor volumes of control mice.

Starting at age 3 months, MMTV-Cre *BRCA1 *^*flox/flox *^*p53*^+/- ^mice were treated with either the EGFR inhibitor erlotinib at 100 mg/kg/day orally (treatment cohort) or vehicle control (control cohort) as dosed previously [35]. End points were tumor-free survival and tolerability of the prophylactic erlotinib treatments. The mice tolerated the treatments well, with the only adverse effect being partial alopecia in about 30% of the mice. Mice were examined daily, and tumors were diagnosed by palpation. Upon necropsy, tumors were counted, fixed and examined for ER expression. Survival analysis (Figure 7) showed a median disease-free survival of 365 days in the erlotinib-treated cohort versus 256 days in the control cohort, that is, erlotinib treatments delayed tumor development by an average of 3 months. Only 19 tumors were observed in the erlotinib-treated cohort versus 31 tumors in the control cohort, a significant reduction (*P *= 0.0003). Upon necropsy, tumors were fixed and processed for immunohistochemistry (Table 1 and Additional file 2, Figure S2). As expected on the basis of previous studies [34,36], the mice in the control cohort developed both ER-positive and ER-negative breast cancers, with a predominance of ER-negative tumors. Interestingly, while the number of ER-positive tumors was not significantly different in both cohorts, the number of ER-negative breast cancers was sharply reduced in the erlotinib-treated cohort (*n *= 5 versus *n *= 19, respectively), indicating that erlotinib was effective in preventing the emergence of ER-negative, but not ER-positive, breast cancers in this mouse model (Table 1). Importantly, EGFR staining showed that the erlotinib-treated cohort had a much lower number of EGFR-positive tumors than the control group, again confirming that erlotinib treatments selected for EGFR-negative tumors (Table 1). ALDH1 staining was observed in nests and at the edges of the tumors in clusters (Additional file 2, Figure S2) and was highly variable among tumors. There was a trend toward lower ALDH1 expression in the erlotinib-treated cohort; however, given the high variability of ALDH1 expression, statistical significance was not reached. The Ki-67 labeling index as a marker for proliferation [37] was also highly variable between tumors and did not differ significantly between the erlotinib-treated and control cohorts, although there was a trend toward higher Ki-67 expression in control tumors (Table 1). The cell death index as assessed by cleaved caspase 3 expression [37] was less variable, and we found a higher cell death index in the erlotinib-treated cohort than in controls, possibly indicating that a fraction of the tumor cells still responded to EGFR inhibition while the majority of tumor cells were resistant. Finally, we examined whether erlotinib had any effect on the growth of established tumors in this mouse model (Figure 7B). Tumor metrics showed that once tumors were established, erlotinib did not shrink these tumors, and tumors grew similarly to the vehicle control-treated tumors. The lack of efficacy of erlotinib on established tumors was seen in ER-negative and ER-positive tumors, further confirming that EGFR inhibition prevented the emergence of ER-negative tumors but likely did not kill nascent ER-negative tumors. In summary, we found that tumors that emerged in erlotinib-treated mice tended to be positive for ER and negative for EGFR and ALDH1. Once tumors were established, their growth was not delayed by treatments with erlotinib, indicating that the majority of tumor cells are resistant to erlotinib treatment and grow independently of EGFR signaling.

**Table 1:** Clinicopathologic features of observed tumors in erlotinib-treated prevention cohort and controls^a^

Clinicopathologic features	Erlotinib (*N *= 13)	Control (*N *= 14)	*P *value
Median disease-free survival, days	365	256	0.0001
Number of tumors	19	31	0.0003
ER-positive tumors	14	12	n.s.
ER-negative tumors	5	19	0.0000002
EGFR^b^			0.0004
0	10	3	
1+	6	8	
2+	0	6	
3+	0	2	
Mean ALDH1^b^, % (±SD)	3.8% (2.8%)	9.0% (9.8%)	0.11 (n.s.)
Mean Ki-67^b^, % (±SD)	21.4% (14%)	30.4% (17.7%)	0.6 (n.s.)
Mean cleaved caspase 3^b^, % (±SD)	18.4% (8.6%)	10.6% (8.4%)	0.018

^a^ER, estrogen receptor; n.s., not significant; EGFR, epidermal growth factor receptor; ALDH1, aldehyde dehydrogenase 1; SD, standard deviation; ^b^only 16 tumors in the erlotinib-treated cohort and 19 tumors in the control cohort could be processed for EGFR, ALDH1, cleaved caspase 3 and Ki-67. A two-sided *t*-test was performed to analyze statistical significance.

## Discussion

### Haploinsufficiency phenotype of *BRCA1 *includes enhanced proliferation of MECs

We previously found that that the nonmalignant MECs from *BRCA1 *mutation carriers contain a subpopulation of progenitor cells with significantly increased clonal and proliferative potential compared with normal controls [8]. Of these cells, 79% had not undergone loss of heterozygosity but had remained heterozygous for *BRCA1 *(retention of heterozygosity), and these cells tended to differentiate into ER-negative, EGFR-positive colonies compared to controls. Our observations confirm that even partial loss of *BRCA1 *leads to an increase a MECs' clonal proliferation (Figures 5 and 6), lending further support to the concept that haploinsufficiency of *BRCA1 *with reduced protein levels of *BRCA1 *leads to a differentiation block coupled with enhanced proliferation of MECs [8,38].

### *BRCA1 *wt and *BRCA1*-haploinsufficient MECs depend on EGFR for proliferation

MECs rely on EGFR activation for migration, proliferation and survival of mammary epithelial progenitor cells. However, the role that EGFR plays in either the initiation or the maintenance of the malignant phenotype is largely unknown. Regardless of whether the progenitor cell population expanded through the loss of *BRCA1 *is defined by expression of ALDH1 [8,29] or Epcam^+^/CD49^+ ^[31], the progenitor cell population expanded in *BRCA1 *mutation carriers shows high EGFR expression relative to the control cells [8,31]. Here we show that suppression of *BRCA1 *leads directly to an increase in EGFR expression with increased clonal growth of MECs (Figure 5), which can be entirely suppressed by the EGFR inhibitor erlotinib (Figures 5 to 7), suggesting that while loss of *BRCA1 *leads to an increase in EGFR activity, loss of *BRCA1 *does not convey growth factor independence.

### Multiple mechanisms contribute to the *BRCA1*-related increase in EGFR expression

A direct regulatory role of *BRCA1 *for the transcription of a receptor tyrosine kinase has been reported for the IGF-IR gene [20,21,39]. Abramovitch *et al. *[17] and Maor *et al. *[18] found that IGF-IR and IGF-IIR mRNA expression levels are elevated in the tissues of women with a genetic predisposition to breast cancer. They showed that *BRCA1 *interacts with and prevents the binding of the specificity protein 1 (Sp1) transcription factor to the IGF-IR receptor. Sp1 is a general transcription factor with a wide range of target promoters, with EGFR being among them [40]. Our data show that downregulation of *BRCA1 *directly increased EGFR mRNA as well as EGFR promoter activity, suggesting transcriptional regulation (Figures 2A and 2B). Whether the regulation of EGFR transcription is also mediated by binding of *BRCA1 *to Sp1 is currently unclear. In addition, we have shown a posttranslational effect of *BRCA1 *on EGFR protein stability (Figures 2C and 2D). The fact that two independent mechanisms converge to increase cellular EGFR levels after *BRCA1 *inhibition suggests the functional importance of this regulatory axis. *BRCA1 *levels fluctuate throughout the cell cycle, and they are highest during the S phase and mitosis [41]. Downstream signaling from EGFR, however, is tightly suppressed during mitosis, as tyrosine phosphorylation of EGFR is highest in the G_0_/G_1 _phase, then gradually decreases during the S and G_2 _phases and reaches its lowest levels during the M phase [42]. Negative regulation of EGFR by *BRCA1 *would ensure the temporal separation between phases when demand for mitogenic signaling is high, that is, G_0_/G_1_, and between phases when mitogenic signaling might interfere with DNA synthesis and repair, that is, the S phase. Such regulatory loops might be dysfunctional in MECs that have lost one or both alleles of *BRCA1*, allowing for an increase in mitogenic signaling of MECs with inherent genetic instability and increased vulnerability to oncogenic transformation. In this scenario, the primary effects of loss of *BRCA1*, that is, an increase in genetic instability, would cooperate with the secondary effect, an increase in EGFR signaling, toward proliferation and eventual transformation of cells with increased genetic instability.

This BRCA1-EGFR cooperation concept could potentially be broadly applicable to mitogenic signaling and might explain why not only EGFR but also IGF-IR [43] is increased in MECs that have lost *BRCA1*. It may also explain why *BRCA1 *has a negative regulatory effect on the stability of phosphorylated Akt [24] and attenuates extracellular signal-regulated kinase activation in response to estrogen or EGF stimulation [44,45]. The hypothesis that even heterozygotic loss of *BRCA1 *may allow for an increase in mitogenic signaling and thereby convey a growth advantage to MECs with genetic instability is further supported by the fact that *BRCA1 *mutation carriers have a strikingly high frequency of atypical ductal hyperplasia (38% in *BRCA1 *carriers versus 4% in control tissues) and ductal carcinoma *in situ *(13% in *BRCA1 *carriers versus none in control tissues) [46], which most often is negative for ER and positive for EGFR [47].

### EGFR inhibition is effective for the prevention but not for the treatment of *BRCA1*-related breast cancers

The expression of EGFR in breast cancer has been linked to endocrine resistance and poor outcomes [48-50]. It has also been postulated that EGFR activation may be an important step in the progression to estrogen independence [51]. EGFR overexpression appears to correlate with the basaloid phenotype and is found in 67% of *BRCA1*-related cancers versus only 18% of non-*BRCA1*-related breast cancers [6]. These findings have prompted the launching of several clinical trials to examine the therapeutic efficacy of the EGF inhibitors gefitinib and erlotinib in ER-negative breast cancer. Early outcome data do not point toward major activity of EGFR inhibitors in unselected patients with metastatic breast cancer [52]. Similarly, presurgical exposure studies have shown only modest or no activity of erlotinib on the proliferative index of TNBCs [53]. Our studies confirm that while erlotinib prevents the emergence of TNBCs, manifest breast tumors grow independently of EGFR signaling (Figure 7B).

### EGFR inhibition prevents the emergence of ER-negative but not of ER-positive breast cancers in *BRCA1*-mutant mice

Currently, there is a lack of nonsurgical primary prevention options for women at risk for TNBC. Our data show that the EGFR inhibitor erlotinib was effective in the prevention of EGFR^+^/ER^- ^breast cancers, but not EGFR^-^/ER^+ ^breast cancers, in *BRCA1*-mutant mice (Figure 7 and Table 1). We have thereby demonstrated for the first time the principle that EGFR inhibition is effective in preventing *BRCA1*-related tumors. The concept of breast cancer prevention through EGFR inhibition has been explored previously; in fact, EGFR inhibitors have been successfully used for the prevention of breast cancer in experimental mouse models [54-57]. However, these mice were *BRCA1*-proficient and at risk for breast cancer because of overexpression of transgenic erbB2 (Her2), which is a member of the EGFR family and a direct target for the drugs used in those studies, that is, lapatinib or gefitinib. However, in humans, erbB2 amplification is the result of a somatic mutation. Thus, it is currently not possible to identify women at risk for the development of Her2-positive breast cancer, thereby limiting the applicability of these data. On the other hand, there is a need to develop medicinal therapeutic strategies for the prevention of TNBC, especially in *BRCA1 *mutation carriers, and our mouse model data suggest that targeting the EGFR pathway might be promising. While erlotinib has a relatively benign toxicity profile, the expected dermatological complications [58] and unknown long-term effects will likely still make it prohibitive to use this particular drug for preventive purposes without time limits. An as yet unsolved question is whether a shorter, limited time period of EGFR inhibition would be protective beyond the actual treatment time, and we are planning to address this issue in this mouse model. However, as increasingly naturally occurring compounds that suppress EGFR signaling are discovered, substances such as allophycocyanins might hold promise for use as chemopreventive agents [59,60]. Our studies suggest that the window of opportunity for effective breast cancer prevention using EGFR inhibitors is a state at which loss of *BRCA1 *and gain of EGFR have occurred, but the growth factor independence of cancer cells has not yet been established.

## Conclusions

We have identified a cooperative effect of loss of *BRCA1 *with gain of EGFR expression that leads to increased clonal proliferation of MECs and may render these cells vulnerable to malignant transformation. This cooperative effect is achieved by transcriptional upregulation as well as posttranslational stabilization of EGFR upon *BRCA1 *downregulation. In addition, cells with loss of *BRCA1 *are enriched for the highly EGFR-expressing ALDH1-positive population. The tumorigenic effect of the cooperation of loss of *BRCA1 *with gain of EGFR in nonmalignant MECs can be disrupted by the preventive use of the EGFR inhibitor erlotinib. Thus, at the premalignant stage, EGFR inhibition may provide a window of opportunity for breast cancer prevention.

## Abbreviations

3D: three-dimensional; ADH: atypical ductal hyperplasia; ALDH1: aldehyde dehydrogenase 1; ATCC: American Type Culture Collection; BAAA: BODIPY aminoacetaldehyde; *BRCA1*: breast cancer gene 1; cDNA: complementary deoxyribonucleic acid; Cre: Cre recombinase; DAPI: 4',6-diamidino-2-phenylindole; DCIS: ductal carcinoma *in situ*; DEAB: diethylaminobenzaldehyde; EGF: epidermal growth factor; EGFR: epidermal growth factor receptor; EpCam: epithelial cell adhesion molecule; ER: estrogen receptor; Erk: extracellular signal-regulated kinase; f: floxed; FBS: fetal bovine serum; GFP: green fluorescent protein; hMEC: human mammary epithelial cell; IGF-IR: type I insulin-like growth factor receptor; IgG2b: immunoglobulin G2b; MEC: mammary epithelial cell; MMTV, mouse mammary tumor virus; mRNA: messenger RNA; MTT: 3-(4,5-dimethylthiazol-2-yl)-2,5-diphenyltetrazolium bromide; PBS: phosphate-buffered saline; PCR: polymerase chain reaction; PE: phycoerythrin; PR: progesterone receptor; Renilla TK: Renilla thymidine kinase; RNA: ribonucleic acid; RT-PCR: real-time polymerase chain reaction; shRNA: small hairpin RNA; siRNA: small interfering RNA; Sp1: specificity protein 1; TNBC: triple-negative breast cancer; wt: wild type.

## Competing interests

The authors declare that they have no competing interests.

## Authors' contributions

GW, LB, HH, AJ and NT developed the concept of and designed the experiments. ST, GW and LB isolated primary MECs. LB and HH performed the cell culture work. GW, LB and AJ performed the mouse studies. GW, HH, LB, AJ and EH analyzed the data. GW, LB and EH wrote the manuscript. All authors read and approved the final manuscript.

## Supplementary Material

Additional file 1 **Figure S1**. Loss of *BRCA1 *leads to an increase in the CD24^low^CD44^high ^stem cell population in mammary epithelial cells (MECs). MCF-10A or human MEC (HMLE) cell lines expressing either control or *BRCA1*-inhibitory small hairpin (shRNA) constructs were examined for CD24 and CD44 expression using dual color flow cytometry. Gates were set using isotype controls for the respective antibodies. Note that the increase in CD24 and loss of CD44 were more pronounced in HMLE cells than in MCF-10 cells. However, in both cell lines, inhibition of *BRCA1 *led to a notable increase in CD24^low^CD44^high ^cells (from 1.1% (control) to 3.8% (sh1) and 8% (sh2) in MCF-10 cells and from 2.6% (control) to 9.2% (sh1) and 11.6% (sh2) in HMLE cells, respectively). Click here for file

Additional file 2 **Figure S2**. Immunohistochemistry of tumors in the erlotinib prevention cohort or controls. Aldehyde dehydrogenase 1 (ALDH1) staining tended to be cytoplasmic and to occur in nests and clusters of cells, as well as at the edges of tumors. Epidermal growth factor receptor (EGFR) staining was seen at the cell membrane and to some extent in the cytoplasm. Estrogen receptor and Ki-67 staining were nuclear, and anti-cleaved caspase 3 antibodies stained cells entirely. Click here for file
