# Supplementary figures and images for: Loss of BRCA1 leads to an increase in epidermal growth factor receptor expression in mammary epithelial cells, and epidermal growth factor receptor inhibition prevents estrogen receptor-negative cancers in BRCA1-mutant mice
Source: Breast Cancer Res. 2011 Mar 11;13(2):R30. doi: 10.1186/bcr2850 (PMC3219192; doi:10.1186/bcr2850)

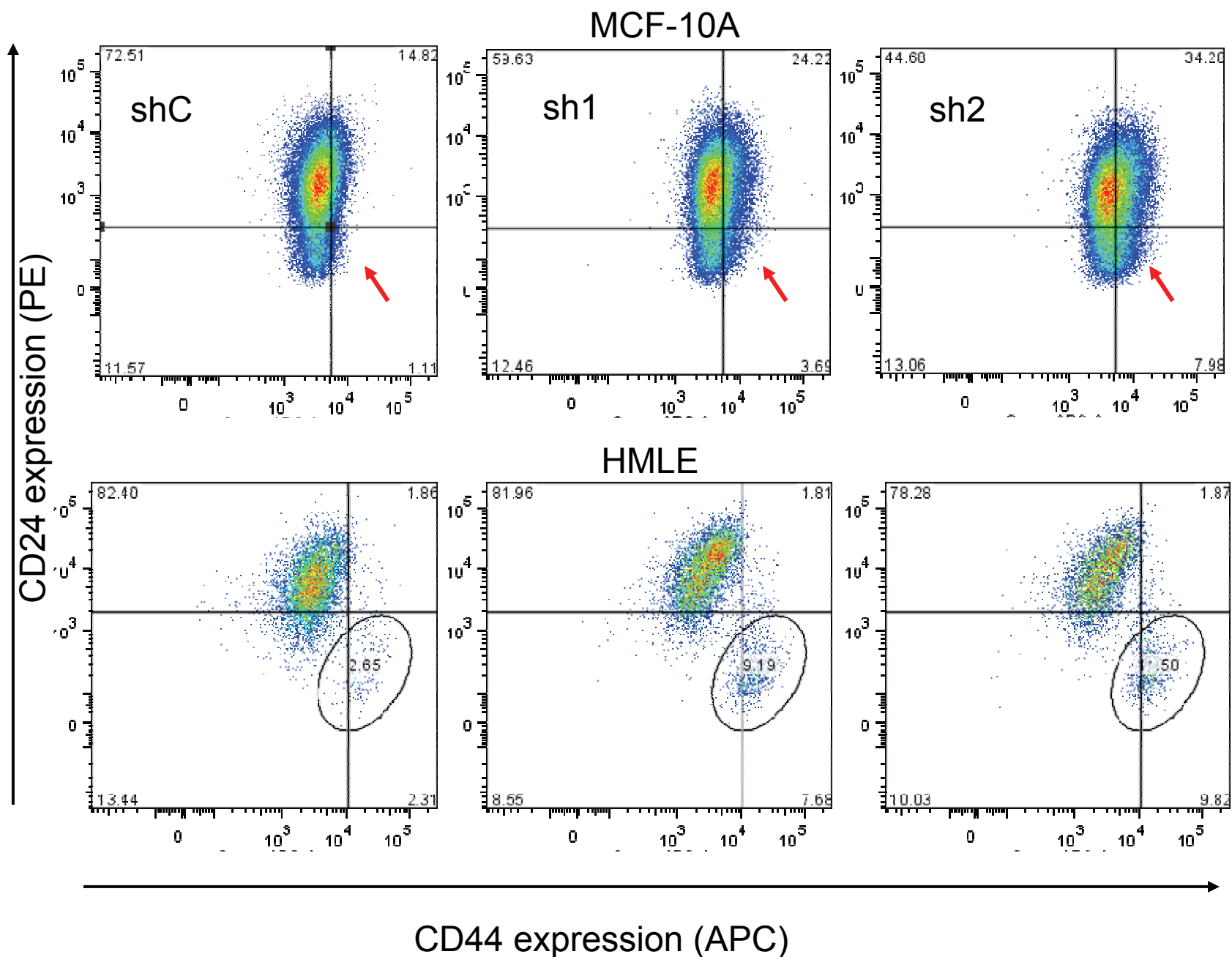

supplemental Fig. 1

Supplement: Additional file 1 — Figure S1. Loss of BRCA1 leads to an increase in the CD24lowCD44high stem cell population in mammary epithelial cells (MECs). MCF-10A or human MEC (HMLE) cell lines expressing either control or BRCA1-inhibitory small hairpin (shRNA) constructs were examined for CD24 and CD44 expression using dual color flow cytometry. Gates were set using isotype controls for the respective antibodies. Note that the increase in CD24 and loss of CD44 were more pronounced in HMLE cells than in MCF-10 cells. However, in both cell lines, inhibition of BRCA1 led to a notable increase in CD24lowCD44high cells (from 1.1% (control) to 3.8% (sh1) and 8% (sh2) in MCF-10 cells and from 2.6% (control) to 9.2% (sh1) and 11.6% (sh2) in HMLE cells, respectively). [file bcr2850-S1.PDF]

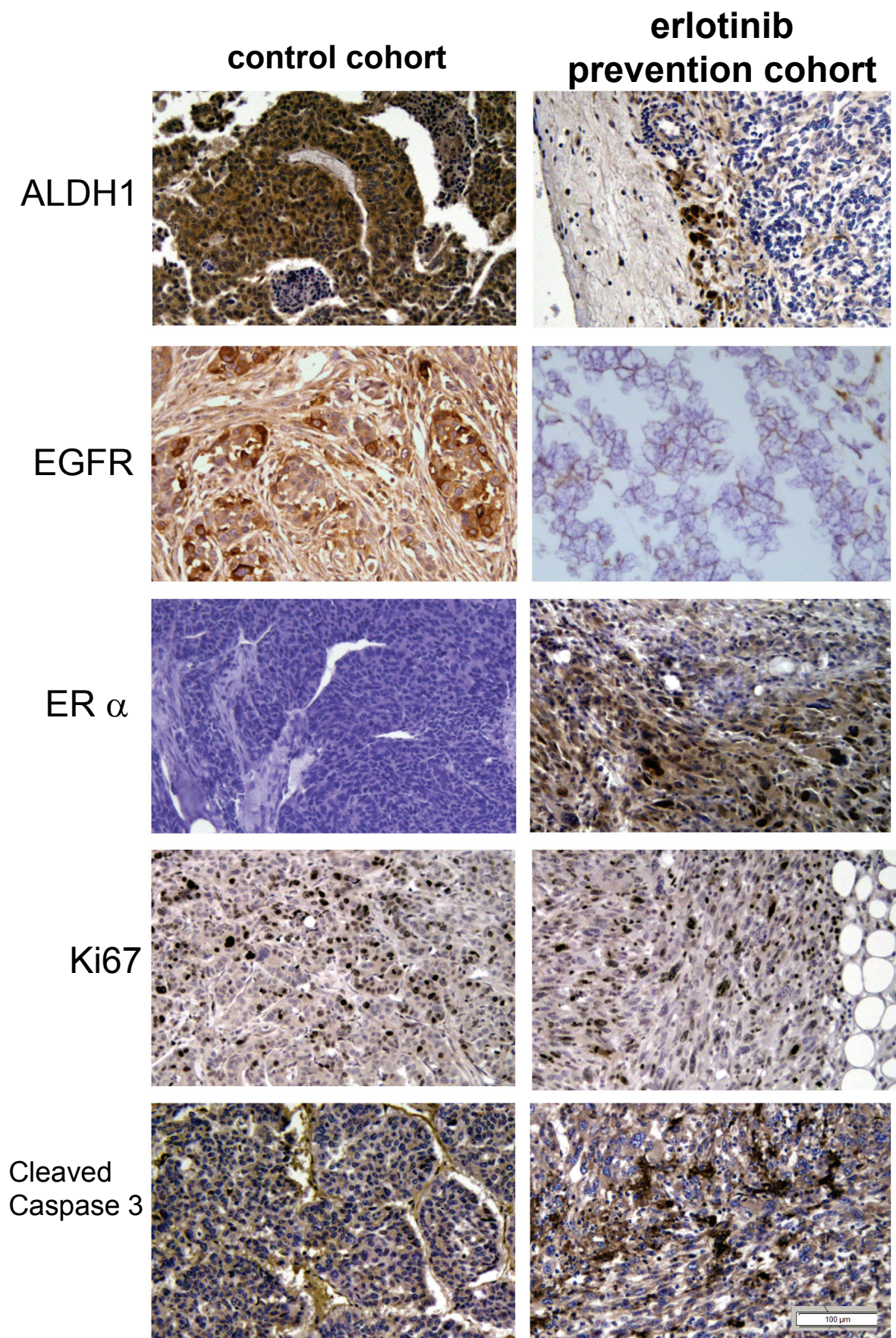

supplemental Figure 2

Supplement: Additional file 2 — Figure S2. Immunohistochemistry of tumors in the erlotinib prevention cohort or controls. Aldehyde dehydrogenase 1 (ALDH1) staining tended to be cytoplasmic and to occur in nests and clusters of cells, as well as at the edges of tumors. Epidermal growth factor receptor (EGFR) staining was seen at the cell membrane and to some extent in the cytoplasm. Estrogen receptor and Ki-67 staining were nuclear, and anti-cleaved caspase 3 antibodies stained cells entirely. [file bcr2850-S2.PDF]
